# Reference Intervals of Serum Metabolites and Lipids of a Healthy Chinese Population Determined by Liquid Chromatography-Mass Spectrometry

**DOI:** 10.3390/metabo15020106

**Published:** 2025-02-07

**Authors:** Yuqing Zhang, Jinhui Zhao, Hui Zhao, Xin Lu, Xueni Jia, Xinjie Zhao, Guowang Xu

**Affiliations:** 1 School of Chemistry, Dalian University of Technology, Dalian 116024, China; yuqingzhang@dicp.ac.cn; 2 CAS Key Laboratory of Separation Science for Analytical Chemistry, Dalian Institute of Chemical Physics, Chinese Academy of Sciences, 457 Zhongshan Road, Dalian 116023, China; zhaojh0714@dicp.ac.cn (J.Z.); luxin001@dicp.ac.cn (X.L.); 3 University of Chinese Academy of Sciences, Beijing 100049, China; 4 Department of the Health Checkup Center, The Second Hospital of Dalian Medical University, Dalian 116023, Chinajxn199901@163.com (X.J.); 5 Liaoning Province Key Laboratory of Metabolomics, Dalian 116023, China

**Keywords:** reference intervals, metabolomics, lipidomics, quantitative analysis, liquid chromatography, mass spectrometry

## Abstract

**Background:** Metabolomics serves as a very useful tool for elucidating disease mechanisms and identifying biomarkers. Establishing reference intervals (RIs) of metabolites in a healthy population is crucial to the application of metabolomics in life sciences and clinics. **Methods:** We enrolled 615 healthy Chinese adults aged between 21 and 85 years. Their health status was ascertained through clinical examinations, biochemical parameters, and medical history. Targeted metabolomics and lipidomics analyses were applied to quantify 705 metabolites and lipids in the serum, establishing RIs and investigating the effect of sex and age on the metabolome and lipidome. **Results:** This study is the first large-scale effort in China to establish RIs for metabolites in the apparently healthy population. We found that most of the sex-related metabolites, including amino acids, acyl-carnitines and triacylglycerols, had higher concentrations in males, while the other sex-related lipids showed higher concentrations in females. Most of the age-related metabolites increased with age, including those associated with protein synthesis, nitric oxide synthesis, energy metabolism, and lipid metabolism. **Conclusions:** This study gives the reference intervals of the healthy Chinese metabolome and lipidome and their relationship with sex and age, which facilitates life sciences and precision medicine, especially for disease research and biomarker discovery.

## 1. Introduction

Blood metabolite concentrations offer valuable insights into physiological and pathological status [1,2]. Metabolomics, the comprehensive analysis of small molecules within biological systems, allows for the detection of hundreds to thousands of metabolites, which presents novel opportunities for characterizing physiological/pathological states and identifying potential biomarkers [3,4]. At present, metabolomics usually identifies biomarkers based on nontargeted analysis; only 2% of the clinical studies combine untargeted and targeted methods [5]. It should be emphasized that an absolute quantification of metabolite concentration changes is very important for the validation of biomarkers and applications in different hospitals [6]. Clinically, according to the Clinical Laboratory and Standards Institute (CLSI) guidelines, the use of diagnostic biomarkers necessitates the establishment of reference intervals (RIs) in a healthy population [7,8]. RIs can be used to describe the expected range of clinical parameters or biomarkers in an apparently healthy population, and the measurement results are compared with the corresponding RIs to determine whether the readouts are normal [8]. RIs are integral to routine clinical testing, playing a crucial role in disease diagnosis and therapeutic decision-making [9,10]. Establishing metabolite RIs in the serum or plasma of healthy individuals is helpful for translating metabolomics research into clinical practice [11] and precision medicine.

The establishment of RIs is typically divided into parametric and non-parametric methods, and many RIs have been established using the latter [12]. In non-parametric approaches, RI limits, including 95% of the reference population, are often set at the 2.5th and 97.5th percentiles [13,14]. In 2022, Cao et al. established the RIs for homocysteine, cysteine, and methionine through a metabolomics analysis of 1553 apparently healthy subjects aged ≥20 years [15]. Homocysteine metabolism has been widely recognized as an independent risk factor for cardiovascular disease (CVD) [16]. Some studies have delineated the “normal” range of serum or plasma metabolites in healthy volunteers [17,18,19]. For instance, Trabado S. et al. established reference values of 144 metabolites in human plasma using data from 800 healthy French volunteers, revealing associations with sex, age, and total blood cholesterol [18]. However, there is a paucity of research employing quantitative methods with comprehensive metabolite coverage to determine metabolite concentrations in the serum of healthy Chinese individuals and to establish corresponding RIs.

**Figure 1:**
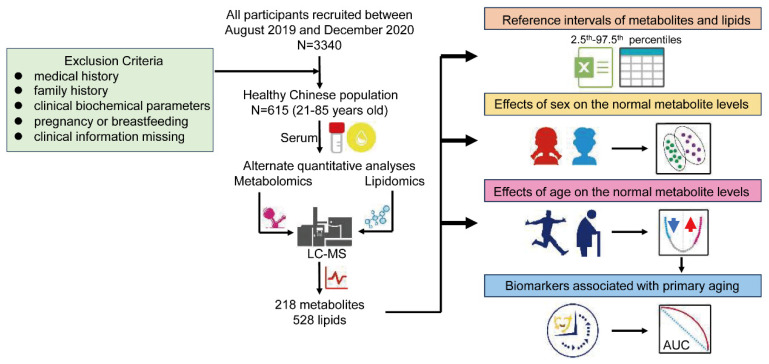
The workflow of the study.

Quantitative metabolomics approaches can be used to obtain absolute concentrations of metabolites and determine the normal metabolic level of “healthy” individuals [20]. Targeted metabolomics is widely recognized as the gold standard for metabolite quantification [21]. In this study, we employed an alternating two-dimensional liquid chromatography system combined with a triple quadrupole mass spectrometer for the targeted detection of metabolites and lipids in the multiple reaction monitoring (MRM) mode [22]. Over 700 metabolites and lipids were quantitatively analyzed in the serum of 615 healthy individuals, and metabolite concentration RIs were established and the influences of sex and age were considered. The research workflow of the research is displayed in Figure 1.

## 2. Materials and Methods

### 2.1. Chemicals

The chromatographic-grade reagents acetonitrile, methanol, and isopropanol utilized were purchased from Merck (Darmstadt, Germany). Ammonium acetate and formic acid were purchased from Fluka ((St. Louis, MO, USA) and J&K Scientific (Beijing, China), respectively. Methyl-tert-butyl ether was purchased from Aladdin (Shanghai, China). Ammonium bicarbonate was obtained from Sigma-Aldrich (St. Louis, MO, USA). Ultrapure water was prepared using a Milli-Q device (Millipore, 7 Billerica, MA, USA). The endogenous metabolite standards were provided by various commercial sources with the corresponding information documented in our previous published paper [22].

### 2.2. Study Cohort and Approval

Participants were recruited from the Second Affiliated Hospital of Dalian Medical University between August 2019 and December 2020. The Ethics Committee of the Second Affiliated Hospital of Dalian Medical University supported this study with approval No. 124, 2019. The human studies were performed according to the principles of the Declaration of Helsinki. All participants provided their informed written consent. It was also agreed that data and information derived from the participants’ serum samples could be utilized for publication.

Recruitment criteria were based on medical history, family history, and clinical biochemical parameters, indicating apparent health. The clinical diagnostic criteria for normal biochemical parameter levels are detailed in Table S1. Exclusion criteria include missing clinical information, pregnancy or lactation, a history of prior surgeries (e.g., thyroidectomy, hysterectomy, hepatectomy, cholecystectomy, pancreatectomy, mastectomy, or nephrectomy), post-surgical ultrasound or computerized tomography findings, familial illness history, obesity, hypertension, hyperuricemia, hyperglycemia, dyslipidemia, or fatty liver diagnosed by ultrasound or computerized tomography, and individuals with diagnosed or suspected severe illnesses such as renal disease, cardiac disease, or cancer. Participants were also excluded if they exhibited more than three abnormal clinical biochemical parameters including waistline, creatinine, blood routine examination (hemoglobin, erythrocyte count, leukocyte count, absolute neutrophil count, absolute lymphocyte count, and platelet count), or liver function test (ALT, AST, GGT, and ALP). Based on these criteria, a total of 615 apparently healthy individuals aged between 21 and 85 were included in the study, 47% of which were male and 53% were female. In this cohort, 73% of the participants had no more than one outlier in their clinical parameters. The detailed clinical characteristics of the 615 participants are listed in Table 1.

### 2.3. Metabolome and Lipidome Extractions and RPLC-MS Analysis

The preparations of the samples were carried out according to the previous method [22]. For metabolome extraction, 50 µL of serum sample was extracted with 200 µL of methanol containing metabolome internal standards (ISs). The detailed information of metabolome ISs is listed in Table S2. After vortexing (2 min) and centrifugation (18,700× *g*, 10 °C, 15 min), the supernatant (100 µL) was lyophilized. The lyophilized samples were reconstituted in 150 µL of acetonitrile/water (2:8, *v*/*v*) for analysis in positive ion mode and 50 µL of methanol/water (2:8, *v*/*v*) for analysis in negative ion mode.

For lipidome extraction, 40 µL of serum sample was mixed with 300 µL methanol containing lipidome ISs (Table S2). Then, 1 mL methyl-tert-butyl ether was added to the mixture and vortexed for 15 min. Next, the mixture was mixed with 300 µL water, vortexed for 30 s, and kept at 4 °C for 10 min. After centrifugation (18,700× *g*, 10 °C, 15 min), 400 µL of the organic phase supernatant was taken for lyophilization. The lyophilized sample was reconstituted in 640 µL or 120 µL of acetonitrile/isopropanol/water (65:30:5 *v*/*v*/*v*, 5 mM ammonium acetate) for positive or negative ion mode analysis.

An alternating two-dimensional reversed-phase liquid chromatography (RPLC) system coupled with a triple quadrupole mass spectrometer (MS) was used for targeted metabolome and lipidome analyses of serum samples [22]. The metabolome and lipidome extracts were separated using two Shimadzu Nexera systems (Shimadzu, Kyoto, Japan); when one system was working, the other was washing and equilibrating.

For metabolome analysis, a BEH C8 column (2.1 × 50 mm, 1.7 µm, Waters, Milford, MA, USA) was employed in positive ion mode, and an HSS T3 column (2.1 × 50 mm, 1.8 µm, Waters, Milford, MA, USA) was used in negative ion mode. Mobile phases consisted of 0.1% formic acid in water (A) and acetonitrile (B) for positive ion mode, and 6.5 mM ammonium bicarbonate in water (A) and methanol/water (95:5) (B) for negative ion mode. The gradient programs were as follows: positive ion mode, 5% B for 0–0.5 min, increasing to 100% B in 8.0 min, then holding at 100% B for 9.0 min; negative ion mode, 2% B for 0–0.5 min, increasing to 100% B in 8.0 min, then holding at 100% B for 9.0 min. Lipidome analyses were performed using 10 mM ammonium acetate in acetonitrile/water (60:40) (A) and isopropanol/acetonitrile (90:10) (B) as mobile phases, with a BEH C8 column in both positive and negative ion modes. The gradient elution was 50% B for 0–1.5 min, increasing to 85% B by 9.0 min.

Autosampler temperatures were set at 4 °C for the metabolome and 10 °C for the lipidome, with flow rates of 0.4 mL min^−1^ and 0.3 mL min^−1^, respectively. Column temperatures were maintained at 60 °C. Data acquisition was performed in multiple reaction monitoring (MRM) mode on an AB QTRAP 6500+ triple quadrupole MS, with parameters as previously reported [22].

The stability of the metabolome and lipidome analysis processes was monitored by using quality control (QC) samples (pooled serum) obtained from our in-house biobank. The metabolome and lipidome extractions of QC samples followed the above processes. The real samples were randomized throughout the analytical sequence, with QC samples evenly inserted among the injections.

### 2.4. Pre-Processing of Data and Statistical Analyses

Raw data were extracted, and peak identification and integration were performed using the MultiQuant software (version 3.0.3; AB SCIEX). The original peak areas of detected metabolites were normalized using their corresponding internal standards. A linear regression model was employed to fit the calibration curve, with weighting based on 1/x^2^. The calibration curve was constructed using a minimum of six concentration points and exhibited a coefficient (R^2^) exceeding 0.99. Missing values were replaced with 1/5 of the minimum positive values of their corresponding metabolites.

The 2.5th and 97.5th percentiles of metabolites and lipids were calculated to determine RIs. Partial least squares discrimination analysis (PLS-DA) of the metabolome and lipidome was performed using SIMCA-P 13.0 (Umetrics, Umea, Sweden). Linear regression models were established to determine the effect of sex and age on metabolite and lipid levels, which were performed on MetaboAnalyst 5.0 (https://www.metaboanalyst.ca accessed on 25 November 2024). Pearson’s correlation coefficients were determined to evaluate the relationship between age and the concentrations of metabolites and lipids. A random forest classifier was used to identify important metabolite and lipid features associated with aging. The random forest method was used to build a diagnostic model after completing the feature selection. The random forest model was implemented using MetaboAnalyst 5.0. All visualizations were conducted using R (v.4.0.4) and GraphPad Prism 8.0.

## 3. Results

### 3.1. Targeted Metabolome and Lipidome Profiling

**Figure 2:**
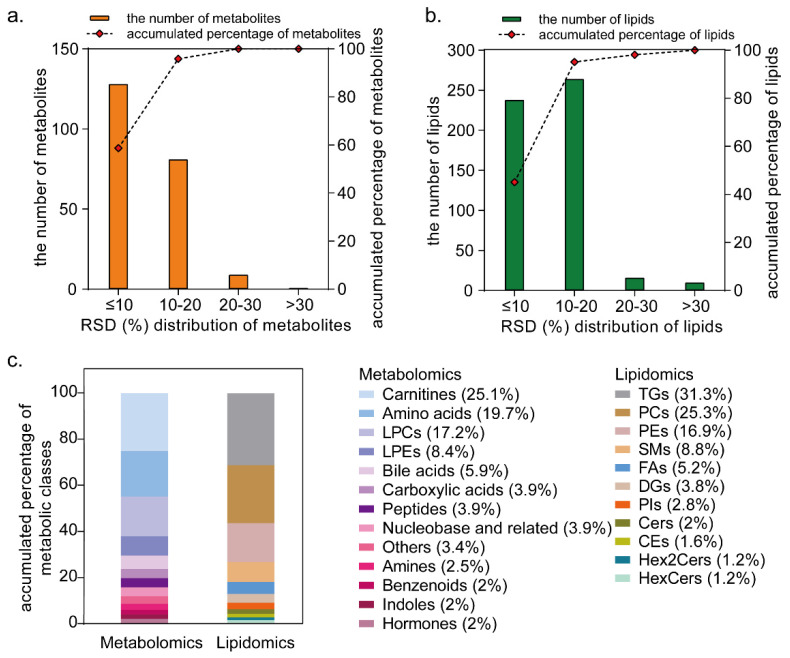
Target profiling of metabolome and lipidome in serum. (**a**,**b**) The distributions of RSD values for metabolites detected by targeted metabolomics and lipidomics of QC samples. (**c**) The classes and proportions of metabolome and lipidome reliably quantified by targeted metabolomics and lipidomics in serum. LPCs: lysophosphatidylcholines, LPEs: lysophosphatidylethanolamines, TGs: triacylglycerols, PCs: phosphatidylcholines, PEs: phosphatidylethanolamines, SMs: sphingomyelins, FAs: fatty acids, DGs: diacylglycerols, PIs: phosphatidylinositols, Cers: ceramides, CEs: cholesterol esters, Hex2Cers: dihexaglycosylceramides, Hexcers: Hexosylceramides.

The close relationship between metabolome and lipidome profiles and health status, as well as metabolic disorders, emphasizes the importance of the concurrent monitoring of metabolite and lipid levels. To achieve precise quantification and enhance metabolite coverage, we employed a targeted metabolomics and lipidomics approach, utilizing an alternating two-dimensional LC separation system coupled with a triple quadrupole MS in MRM mode [22]. We constructed calibration curves using a panel of 124 endogenous metabolite standards to quantify most of the metabolites accurately. A total of 47 isotope-labeled standards or exogenous metabolite standards covering diverse metabolic classes and pathways were used as internal standards to normalize the peak areas. Among these, lipids, carnitines, bile acids, and free fatty acids (FFAs) were quantified using the same classes of endogenous metabolite standards. In total, 218 metabolites and 528 lipids were detected in the serum samples. A total of 711 of them had an RSD ≤ 20% in the QC samples (Figure 2a,b), indicating the stability of the analysis. The proportions of the metabolite and lipid classes are depicted in Figure 2c.

### 3.2. Establishment of Reference Intervals for Metabolites and Lipids

We performed a quantitative analysis of the serum metabolites and lipids in a cohort of 615 healthy Chinese adults. The data of the metabolites and lipids were included if the RSD ≤ 20% in the QC samples and missing values (not detected or concentrations below the limit of detection) was less than 15% in real samples. Consequently, the RIs for 705 metabolites and lipids (2.5th to 97.5th percentiles) were established, including 203 metabolites and 502 lipids; the details are listed in Table S3.

We compared the results of creatinine and triglyceride, which can be detected by both LC-MS and clinical biochemical detection. The results from our LC-MS analysis exhibited a high concordance with conventional biochemical methods within this cohort, as evidenced by the Pearson correlation coefficients for creatinine (r = 0.91) and triglycerides (r = 0.87) (Figure S1a,b). This correlation confirms the accuracy and reliability of our LC-MS measurements.

A previous study established reference values (mean) and intervals (25th–75th percentiles) for 144 metabolites in the plasma of 800 healthy French volunteers [18]. We identified the metabolites in our dataset that corresponded to those reported and compared their reference values and intervals (25th–75th percentiles). The comparison results are shown in Figure S2. Significant differences in the levels of citrulline, phenylalanine, histidine, serine, glycine, butyrylcarnitine, nonaylcarnitine, and propionylcarnitine were observed between the French and Chinese populations. Additionally, LipidQC software [23] was utilized to compare our lipidomics data with previous consensus values from 31 independent laboratories. A total of 102 lipids that were present in both LipidQC and our data were compared (Figure S3b). We noted that the lipid quantification values falling outside the 99% uncertainty region were predominantly lower than the reported values. The differences in the concentration ranges of the metabolites studied may be due to genetic and lifestyle differences [24], which also emphasizes the significance of establishing RIs specific to the Chinese population.

**Figure 3:**
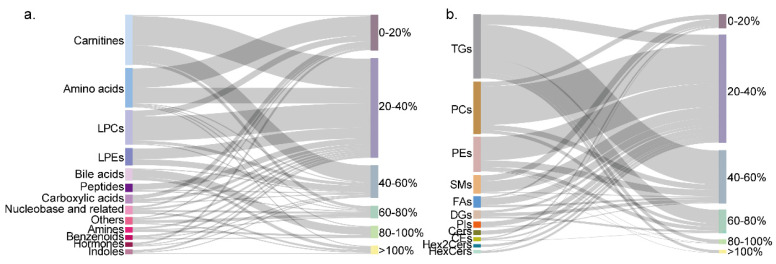
The distributions of CVs of 705 metabolites in serum of an apparently healthy population. (**a**) The distributions of CVs of 203 metabolites in serum. (**b**) The distributions of CVs of 502 lipids in serum.

The individual differences in the serum metabolites and lipids showed varying degrees of fluctuations. In this work, the coefficients of variation (CVs) for metabolites were employed to evaluate the variability in the metabolite concentrations within the RIs. The majority of the metabolites (84.2%) and lipids (80.9%) exhibited CVs below 60% (Figure 3a,b). Notably, the CVs for most amino acids constituting proteins were found to fluctuate within a narrow range, typically less than 20%, including glycine, serine, valine, leucine, histidine, phenylalanine, etc. (Figure 3a). Metabolites associated with energy metabolism, such as most of the fatty acids, acyl-carnitines, phospholipids (PCs, PEs, and SMs), citric acid, and succinic acid, demonstrated CVs below 40% within the RIs. In contrast, over 65% of the TGs and DGs exhibited a significant fluctuation range, with the CVs exceeding 40%. The microbiota-related metabolites, including nearly all the bile acids and indole-3-propionic acid, showed large CVs, exceeding 80% or even 100%. Furthermore, the diet-derived metabolites such as caffeine displayed the highest CVs within the RIs, followed by 1,7-dimethylxanthine. A detailed list of the CVs for the metabolites and lipids in the serum of the apparently healthy Chinese individuals is provided in Table S4.

### 3.3. Effects of Sex on the Metabolome and Lipidome Levels

**Figure 4:**
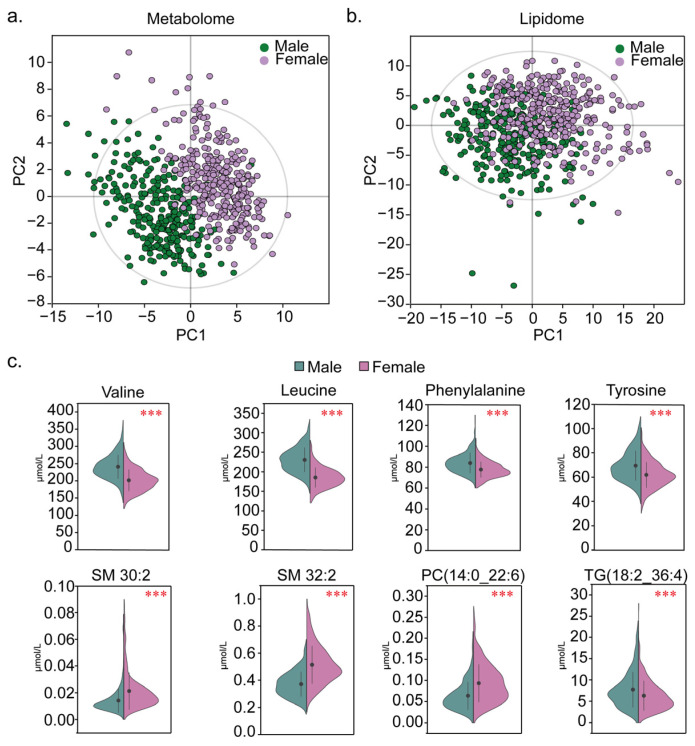
Metabolite concentration difference between male (*n* = 286) and female (*n* = 329) cohorts. (**a**) Partial least squares discrimination analysis (PLS-DA) of metabolite levels in male and female groups; the cross-validation showed no overfitting. (**b**) PLS-DA of lipid levels in male and female groups; the cross-validation showed no overfitting. (**c**) Differential levels of several metabolites and lipids between male and female individuals. The y-axis displays metabolic level. The FDR value (*** < 0.001) from *t*-test is shown. The black dot with line shows the mean ± 1 SD.

The linear regression models were used to adjust for the influence of age and BMI, and significant differences in the concentrations of 99 metabolites and 161 lipids between the male and female subjects were found (*p* < 0.001, Supplementary Table S5). The PLS-DA showed a clear segregation between the sexes in both the metabolome and lipidome profiles (Figure 4a,b). In the serum metabolome, the majority of the metabolites associated with sex exhibited elevated levels in the males (Supplementary Table S5). For example, many amino acids were identified as sex-dependent and were significantly higher in the males (Figure 4c). In the serum lipidome, several sex-associated triglycerides (TGs) demonstrated increased levels in the males (Supplementary Table S5). On the contrary, the concentrations of many free fatty acids and phospholipids were significantly reduced in the males compared to the females (Figure 4c, Supplementary Table S5). Our findings elucidate the presence of sex-specific metabolites and lipids under physiological conditions, highlighting the metabolic sexual dimorphism and suggesting stratification by sex during the establishment of RIs. Herein, we present the sex-specific RIs in Supplementary Table S3.

### 3.4. Effects of Age on the Metabolome and Lipidome Levels

**Figure 5:**
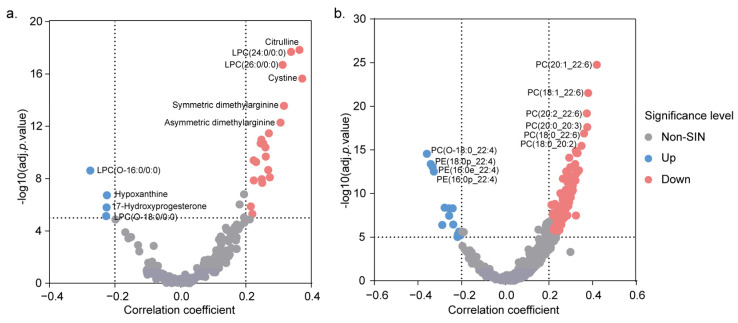
Metabolome and lipidome associated with age. (**a**) A volcano plot depicting age-related changes in metabolites. (**b**) A volcano plot depicting age-related changes in lipids. The y-axis represents the statistical significance of the metabolites associated with age. Linear models adjusted for sex and BMI. Adj. *p* value cut-off was set at a significance level of *p* < 10^−5^. Red dot represents positive correlation and blue for negative correlation. Correlation coefficients between metabolites and age were calculated using Pearson’s method. Correlation coefficient cut-off was set at a significance level of r > 0.2 or r < −0.2 with *p* < 0.05. Top six significantly correlated metabolites and lipids are labeled.

The effects of age on the serum metabolome and lipidome profiles were further investigated. The linear regression models were used to adjust for the influence of sex and BMI, and combined with the correlation analyses, 25 metabolites and 132 lipids that were significantly associated with age were identified (Table S6). In the metabolome dataset, several compounds including cystine, citrulline, symmetric dimethylarginine (SDMA), asymmetric dimethylarginine (ADMA), and various lysophosphatidylcholines (LPCs) and phosphatidylcholines (PCs) demonstrated strong positive correlations with age. Conversely, 17-hydroxyprogesterone (17-OHP); hypoxanthine; and certain ether- or acetal-LPCs, -PCs, and -PEs revealed negative correlations with age (Figure 5a,b). These findings offer comprehensive insight into the alterations in the metabolite and lipid metabolism associated with aging. The age-specific reference intervals are detailed in Supplementary Table S3.

**Figure 6:**
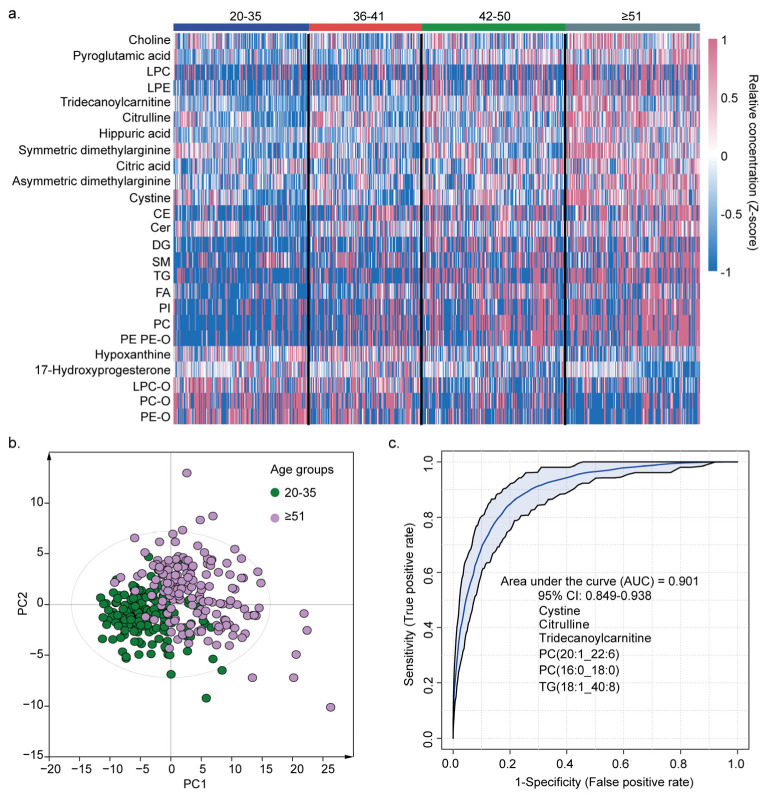
Identification of biomarkers associated with physiological aging. (**a**) Metabolic maps of four different age groups. Metabolome and lipidome concentrations were normalized using Z-scores. (**b**) PLS-DA score plot of metabolome and lipidome data on young (age 20–35) and older (age ≥ 51) groups; the cross-validation showed no overfitting. (**c**) ROC curve of the biomarker panel designed to distinguish between young (age 20–35) and older (age ≥ 51) groups. AUC and the 95% confidence interval of the model are shown in the diagram.

We stratified the study population into four age quartiles to investigate the metabolic changes associated with physiological aging. Heat maps were constructed to demonstrate the alterations in metabolite and lipid levels across these age subgroups (Figure 6a). To enhance the interpretability of the heat maps, metabolites and lipids in the same category that displayed consistent age-related trends were merged together. The PLS-DA clearly differentiated the younger group (21–35 years) from the older group (≥51 years) (Figure 6b). The clinical characteristics of these two groups are detailed in Supplementary Table S7. To identify metabolic markers closely associated with physiological aging, we employed a random forest model for age-related feature selection between the younger and older groups. Six important features (cystine, citrulline, tridecanoylcarnitine, PC(20:1_22:6), PC(16:0_18:0), and TG(18:1_40:8)) were identified and employed to build a random forest classifier to distinguish the young group and older group. The panel of biomarkers effectively discriminated between the younger and older groups, with an area under the receiver operating characteristic curve (AUC) of 0.901 (0.849–0.938) (Figure 6c), indicating a high predictive accuracy for age-related physiological changes.

## 4. Discussion

Utilizing an alternating injection-based metabolomics and lipidomics approach, we established concentration reference intervals for 705 metabolites and lipids. This method has been demonstrated to be stable and reliable for the analysis of a large cohort of samples. To our knowledge, this is the first large-scale study to establish RIs for serum metabolites and lipids in an apparently healthy China population.

The individual differences in the serum metabolite and lipid profiles have a significant impact on the investigation of metabolic dysregulation and the discovery of disease biomarkers. Some metabolites with lower CV values are more likely to be discovered and validated as potential biomarkers. Conversely, metabolites with higher CV values must show greater changes and undergo a rigorous validation process. For example, tryptophan, exhibiting a change rate of 0.87, has been identified and confirmed as a potential biomarker for pediatric cerebral palsy [25]. The bile acid was identified as a biomarker of rectal adenoma [26]; its concentration demonstrated a more than threefold increase over that of the control.

Our findings elucidate the presence of sex-specific metabolites and lipids under physiological conditions. Therefore, the establishment of RIs according to gender is helpful for precise clinical application [27]. We observed an increase in the levels of FFAs, carnitines, and branched-chain amino acids in the male participants. Gender differences in oxidative kinetics during rest and exercise have been suggested [28]. A European cohort study of healthy volunteers demonstrated significantly higher plasma concentrations of leucine, valine, tyrosine, and other amino acids in males [29]. TGs, serving as an energy reservoir, are pivotal in energy metabolism and adenosine triphosphate (ATP) synthesis [30]. A previous investigation supported our findings of elevated TG levels in males [31]. The elevated levels of SMs in females may be linked to estrogen metabolism [32].

Similarly, age also affects the serum metabolome and lipid profile. Cystine is associated with protein biosynthesis, and several studies have reported a significant positive correlation between cystine levels and age, which is consistent with our finding [33,34]. We observed an age-related increase in the serum concentrations of ADMA, SDMA, and citrulline, all of which are involved in the nitric oxide (NO) metabolic pathway. NO is a potent vasodilator and a cardioprotective molecule [35]. Citrulline is involved in NO synthesis [33]. Elevated plasma concentrations of ADMA and SDMA may indirectly affect the activity of nitric oxide synthase (NOS), thereby inhibiting NO production [36]. Studies have indicated that the bioavailability of NO decreases with age [37]. The concentrations of citric acid, propionylcarnitine, and tridecanoylcarnitine increased with age, which are involved in energy metabolism, including the tricarboxylic acid (TCA) cycle and β-oxidation. Cellular energy metabolism is tightly regulated, and its alteration is considered a hallmark of age-related physiological decline [38,39]. The levels of most lipid subclasses increased with age, such as LPCs, PCs, Cers, SMs, TGs, etc., involving multiple aspects such as chronic diseases and cellular aging [40,41,42]. Studies have shown that an increase in LPC levels is associated with cardiovascular and neurodegenerative diseases, and some LPCs are negatively correlated with C-reactive protein increasing with age [41]. However, some LPC-O, PC-O, and PE-O lipids are negatively correlated with age. The study by Beyene et al. revealed that PC-O and PE-O lipids were inversely associated with age, which is consistent with our findings [31]. Decreased levels of PC-O and PE-O lipids have been associated with oxidative stress and age-related metabolic diseases, such as Alzheimer’s disease [43,44]. In our study, the results provide insights into the metabolic features of physiological aging based on an apparently healthy population.

## 5. Conclusions

In this study, we established RIs for serum metabolites in a large-scale cohort of apparently healthy Chinese individuals for the first time using a high-coverage quantitative approach combining metabolomics and lipidomics. This approach ensures the reliability of large-scale sample analysis and facilitates inter-study comparability. These RIs provide benchmarks for metabolite concentrations under physiological homeostasis, which are instrumental for clinical diagnostics and the identification of biomarkers, and helpful for precision medicine. Our findings reveal sex- and age-specific metabolite and lipid profiles, emphasize the necessity of stratification in disease-related research and biomarker identification. The top five metabolites and lipids, including 4-hydroxyphenyllactic acid, creatinine, Glu-Leu, leucine, and SM 32:2, exhibited significant associations with sex. Additionally, the top five metabolites and lipids-citrulline, LPC(24:0/0:0), PC(18:1_22:6), PC(20:1_22:6), and PC(20:2_22:6) showed significant associations with age. Furthermore, the identification of age-related metabolites provides valuable insights into the metabolic changes associated with physiological senescence. In conclusion, the establishment of RIs for healthy Chinese individuals is important for advancing clinical research and applications, thereby enabling the realization of precision medicine.

## Figures and Tables

**Table 1:** Clinical characteristics of the healthy population.

Characteristics	Total	Male	Female
Number	615	286	329
Age (years) (SD)	43.5 ± 11.3	44.5 ± 12.3	42.5 ± 10.3
Waistline (cm) (SD)	78.3 ± 8.1	82.9 ± 6.8	74.3 ± 6.8
Body mass index (Kg/m^2^) (SD)	22.5 ± 2.4	23.2 ± 2.3	21.8 ± 2.3
Systematic blood pressure (mmHg) (SD)	118.4 ± 10.8	120.9 ± 10.1	116.2 ± 10.9
Diastolic blood pressure (mmHg) (SD)	72 ± 8.1	74 ± 7.4	70.2 ± 8.2
Creatinine (µmol/L) (SD)	67.1 ± 14.1	79 ± 9.7	56.7 ± 7.6
Uric acid (µmol/L) (SD)	308.7 ± 62.2	352.8 ± 45.4	270.5 ± 47.9
Blood glucose (mmol/L) (SD)	5.3 ± 0.3	5.4 ± 0.3	5.2 ± 0.3
Total cholesterol (mmol/L) (SD)	4.7 ± 0.7	4.6 ± 0.7	4.7 ± 0.7
Triglyceride (mmol/L) (SD)	1.2 ± 0.4	1.3 ± 0.4	1.1 ± 0.4
HDL-cholesterol (mmol/L) (SD)	1.5 ± 0.3	1.4 ± 0.3	1.6 ± 0.3
LDL-cholesterol (mmol/L) (SD)	2.5 ± 0.6	2.5 ± 0.6	2.4 ± 0.6
Hemoglobin (g/L) (SD)	142.1 ± 15.4	154.2 ± 9.2	131.7 ± 11.5
Erythrocyte count (10^12^/L) (SD)	4.7 ± 0.4	5.1 ± 0.4	4.5 ± 0.3
Leukocyte count (10^9^/L) (SD)	5.5 ± 1.3	5.7 ± 1.4	5.4 ± 1.2
Absolute neutrophil count (10^9^/L) (SD)	3.3 ± 1.0	3.3 ± 1.0	3.3 ± 1.0
Absolute lymphocyte count (10^9^/L) (SD)	1.8 ± 0.5	1.9 ± 0.5	1.7 ± 0.5
Platelet count (10^9^/L) (SD)	236 ± 52.3	222.4 ± 46.2	247.8 ± 54.5
Alanine aminotransferase (U/L) (SD)	18.9 ± 8.4	21.5 ± 9.1	16.7 ± 7.1
Aspartate aminotransferase (U/L) (SD)	20.6 ± 5.2	21.4 ± 4.9	20 ± 5.4
γ-Glutamyl transferase (U/L) (SD)	16.2 ± 9.1	19.6 ± 10.2	13.2 ± 6.9
Alkaline phosphatase (U/L) (SD)	56.3 ± 14.4	60.3 ± 15.0	52.9 ± 13.0

## Data Availability

The data which are made available have been provided in the Supplementary Materials.

## Supplementary Materials

The following supporting information can be downloaded at: https://www.mdpi.com/article/10.3390/metabo15020106/s1, Table S1: Criteria for biochemical examination. Table S2: Detailed information of metabolome and lipidome internal standards. Table S3: Reference intervals of metabolite concentrations (µM) in serum. Table S4: The CV values of metabolites and lipids in serum from apparently healthy individuals. Table S5: Sex-related metabolites and lipids. Table S6: Age-related metabolites and lipids. Table S7: Clinical characteristics of young and older groups. Figure S1: Comparison of metabolite concentrations between biochemical test and LC-MS platform. Figure S2: Comparison of reference values (mean value) and intervals (25th–75th percentiles) of metabolite concentrations derived from our experimental data and the literature. Figure S3: Comparison of metabolite concentrations with SRM 1950.
