# Supplementary material for: Reference Intervals of Serum Metabolites and Lipids of a Healthy Chinese Population Determined by Liquid Chromatography-Mass Spectrometry
Source: Metabolites. 2025 Feb 7;15(2):106. doi: 10.3390/metabo15020106 (PMC11857409; doi:10.3390/metabo15020106)
Supplement: Supplementary file 1 [file metabolites-15-00106-s001.zip › Supplementary material.pdf]

# Reference intervals of serum metabolites and lipids of the healthy Chinese population determined by liquid chromatography-mass spectrometry

Yuqing Zhang[1, 2], Jinhui Zhao[2, 3], Hui Zhao[4], Xin Lu[2, 5], Xueni Jia[4], Xinjie Zhao[2, 5,*], Guowang Xu[1, 2, 3, 5,*]

[1] School of Chemistry, Dalian University of Technology, Dalian 116024, P. R. China.

[2] CAS Key Laboratory of Separation Science for Analytical Chemistry, Dalian Institute of Chemical Physics, Chinese Academy of Sciences, 457 Zhongshan Road, Dalian 116023, P. R. China.

[3] University of Chinese Academy of Science, Beijing, 100049, P. R. China.

[4] Department of the health checkup center, The Second Hospital of Dalian Medical University, Dalian 116023, P. R. China.

[5] Liaoning Province Key Laboratory of Metabolomics, Dalian 116023, P. R. China.

* Correspondence: xj_zhao1@126.com (X. Zhao); xugw@dicp.ac.cn (G. Xu)

**List of Supporting Information**

Table S1 Criteria for biochemical examination.

Table S2 Detailed information of metabolome and lipidome internal standards.

Table S3 Reference intervals of metabolite concentrations (μM) in serum.

Table S4 The CV values of metabolites and lipids in the serum from apparently healthy individuals.

Table S5. Sex-related metabolites and lipids.

Table S6 Age-related metabolites and lipids.

Table S7 Clinical characteristics of young and older groups.

Tables S1-S7 are provided in separated excel sheet.

Figure S1 Comparison of metabolite concentrations between biochemical test and LC-MS platform.

Figure S2 Comparison of reference values (mean value) and intervals (25th - 75th percentiles) of metabolite concentrations derived from our experimental data and the literature.

Figure S3 Comparison of metabolites concentrations with SRM 1950.

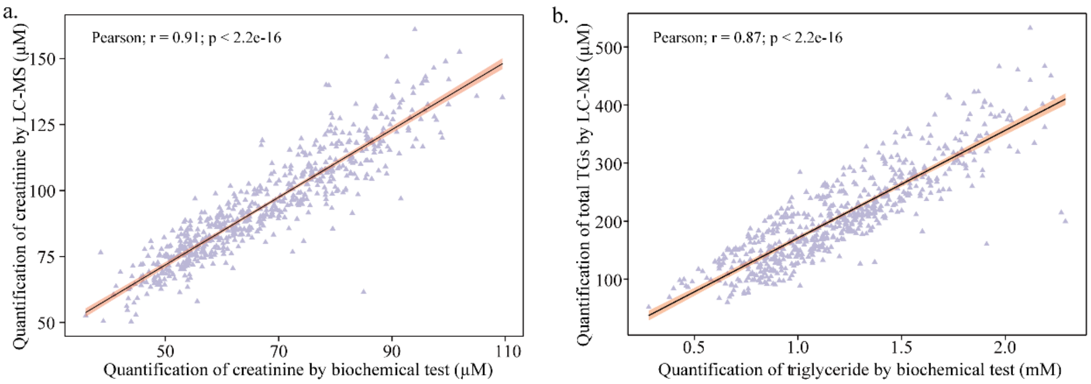


Figure. S1 Comparison of metabolite concentrations between biochemical test and LC-MS platform. a. The correlation of creatinine concentration between biochemical test and LC-MS platform. b. The correlation of total TG concentration between biochemical test and LC-MS platform. The x-axis shows the metabolic concentration determined by biochemical test. The y-axis shows the metabolic concentration determined by LC-MS. Each triangle represents one sample. The line represents the best fit line of linear regression with 95% confidence intervals (CIs). The Pearson correlation coefficient and its corresponding p value are presented.

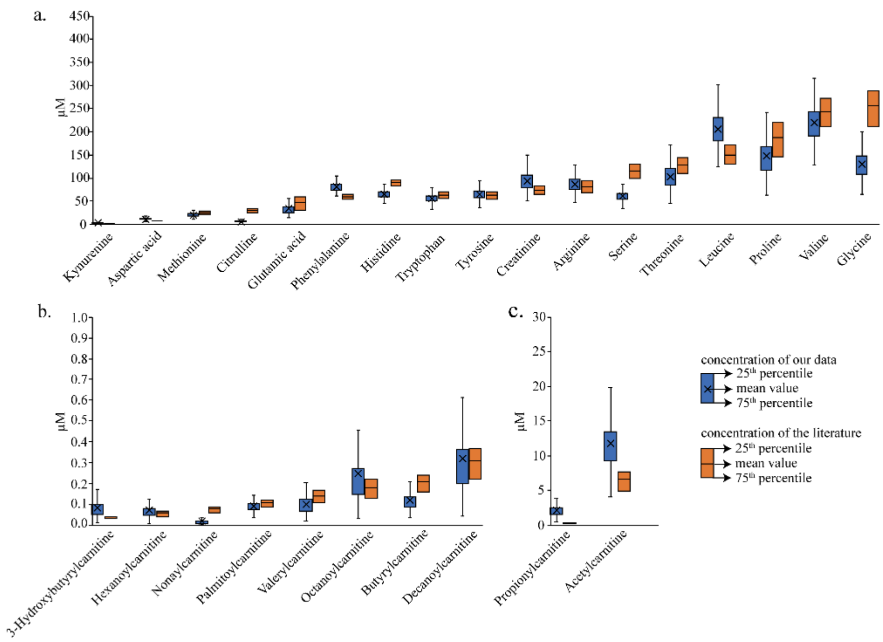


Figure. S2 Comparison of reference values (mean value) and intervals (25th - 75th percentiles) of metabolite concentrations derived from our experimental data and the literature. a. Comparison of amino acid class concentrations between our experimental data and the literature. b-c. Comparison of acylcarnitine class concentrations between our experimental data and the literature.

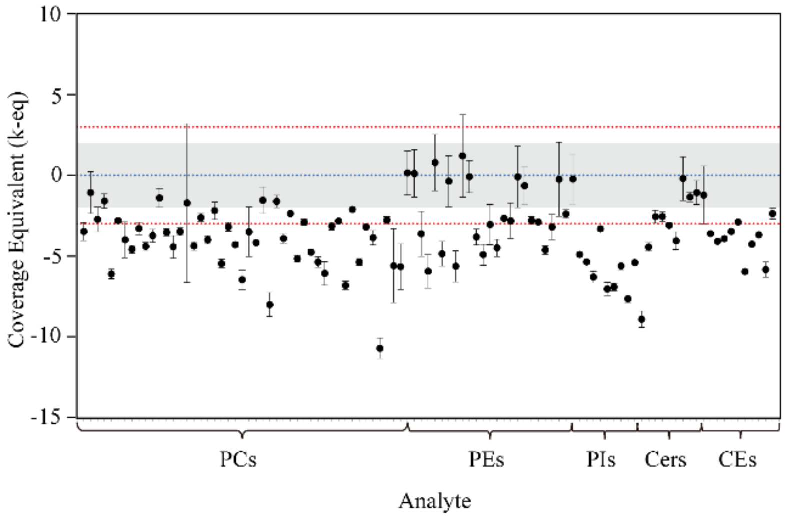


Figure. S3 Comparison of our lipidomics data with previous consensus values from 31 independent laboratories. Our experimental values are represented in measured mean (points) and standard deviation (error bars), overlaid on consensus mean (blue line) and uncertainty (95% coverage-gray area, 99% coverage-red area).
